# Co-culture of osteochondral explants and synovial membrane as *in vitro* model for osteoarthritis

**DOI:** 10.1371/journal.pone.0214709

**Published:** 2019-04-02

**Authors:** Eva Haltmayer, Iris Ribitsch, Simone Gabner, Julie Rosser, Sinan Gueltekin, Johannes Peham, Ulrich Giese, Marlies Dolezal, Monika Egerbacher, Florien Jenner

**Affiliations:** 1 Department for Companion Animals and Horses, University Equine Hospital, Equine Surgery, University of Veterinary Medicine, Vienna, Austria; 2 Department of Pathobiology, Histology and Embryology, University of Veterinary Medicine, Vienna, Austria; 3 Institute of Applied Synthetic Chemistry, Technical University, Vienna, Austria; 4 Molecular Diagnostics, Center for Health and Bioresources, AIT Austrian Institute of Technology, Vienna, Austria; 5 Department of Biomedical Sciences, Bioinformatics and Biostatistics Platform, University of Veterinary Medicine, Vienna, Austria; Dasman Diabetes Institute, KUWAIT

## Abstract

The purpose of the current study was to establish an *in vitro* model for osteoarthritis (OA) by co-culture of osteochondral and synovial membrane explants. Osteochondral explants were cultured alone (control-1) or in co-culture with synovial membrane explants (control-2) in standard culture medium or with interleukin-1β (IL1β) and tumor necrosis factor (TNFα) added to the culture medium (OA-model-1 = osteochondral explant; OA-model-2 = osteochondroal-synovial explant). In addition, in OA-model groups a 2-mm partial-thickness defect was created in the centre of the cartilage explant. Changes in the expression of extracellular matrix (ECM) genes (collagen type-1 (Col1), Col2, Col10 and aggrecan) as well as presence and quantity of inflammatory marker genes (IL6, matrix metalloproteinase-1 (MMP1), MMP3, MMP13, a disintegrin and metalloproteinase with-thrombospondin-motif-5 (ADAMTS5) were analysed by immunohistochemistry, qPCR and ELISA. To monitor the activity of classically-activated pro-inflammatory (M1) versus alternatively-activated anti-inflammatory/repair (M2) synovial macrophages, the nitric oxide/urea ratio in the supernatant of osteochondral-synovial explant co-cultures was determined. In both OA-model groups immunohistochemistry and qPCR showed a significantly increased expression of MMPs and IL6 compared to their respective control group. ELISA results confirmed a statistically significant increase in MMP1and MMP3 production over the culturing period. In the osteochondral-synovial explant co-culture OA-model the nitric oxide/urea ratio was increased compared to the control group, indicating a shift toward M1 synovial macrophages. In summary, chemical damage (TNFα, IL1β) in combination with a partial-thickness cartilage defect elicits an inflammatory response similar to naturally occurring OA in osteochondral explants with and without osteochondral-synovial explant co-cultures and OA-model-2 showing a closer approximation of OA due to the additional shift of synovial macrophages toward the pro-inflammatory M1 phenotype.

## Introduction

Osteoarthritis (OA), a chronic degenerative joint disease characterized by cartilage breakdown, subchondral bone remodeling and synovial inflammation, is the most common musculoskeletal disorder in humans as well as in horses. Secondary to a variety of etiologic factors such as mechanical injury, genetics, ageing, gender and obesity, a common molecular pathway linking biochemical and biomechanical processes leads to the typical pathological progression of OA with an imbalance of cartilage matrix synthesis and degradation and a vicious positive feedback loop involving cartilage breakdown and synovial inflammation [1–11]. During initiation and progression of OA, inflammatory and catabolic mediators are released by cartilage, subchondral bone and synovium, which communicate via cell–cell interactions, through the release of soluble mediators and via mechanical signals [6–11]. In the synovial membrane, macrophages represent the key effector cells guiding synovial inflammation and show significantly rising cell numbers with increasing inflammation grade [12–18]. The synovial macrophages can be classified as “classically activated” M1 macrophages that have a pro-inflammatory phenotype and “alternatively activated” M2 macrophages with an anti-inflammatory phenotype and involvement in tissue remodeling [12–18] The two macrophage phenotypes can be distinguished by their arginine metabolism into nitric oxide (NO) and citrulline (M1 macrophages) or ornithine and urea (M2 macrophages). Hence the NO/urea ratio reflects the M1/M2 polarization and can be used as a functional readout of their relative proportions [19, 20].

The two major cytokines involved in the pathogenesis of OA, interleukin-1β (IL1β) and tumor necrosis factor alpha (TNFα) are mainly produced by M1 synovial macrophages, which thus seem to drive the inflammatory and destructive responses of the synovial fibroblasts [6, 9, 21]. These 2 cytokines synergistically activate other major pro-inflammatory mediators (IL6, IL10, prostglandin F2) and proteolytic enzymes (matrix metalloproteinase-1 (MMP1), MMP3, MMP13, a disintegrin and metalloproteinase with thrombospondin motif-4 (ADAMTS4) and ADAMTS5) throughout the joint [22, 23]. In addition to contributing to cartilage breakdown, the inflamed synovium also plays a major role in the osteoclastogenesis of subchondral bone in OA. Subchondral bone in turn also plays a dual role in the OA process, as a source of inflammatory mediators implicated in clinical OA pain, hypertrophic differentiation of chondrocytes and in the degradation of the deep layer of cartilage as well as through abnormal stress distribution of the bone-cartilage interface secondary to sclerosis and remodeling of the subchondral bone [9, 24–27]. Unfortunately, to date no disease-modifying therapies are available to regenerate degraded hyaline cartilage or decelerate disease progression. Elucidating the common molecular switches regulating this complex multifactorial disease would facilitate the development of targeted therapies.

While *in vivo* trials using animals suffering from naturally occurring OA most accurately reflect the disease, *in vitro* models allow for systematic analysis of various cellular, biophysical and biochemical cues at a high level of standardisation in a controlled environment, without the natural variability found in animal models. *In vitro* models are well suited to study the pathophysiology of diseases and evaluate new therapeutic approaches, while reducing and replacing animal trials in accordance with the three R’s principle. Although a variety of *in vitro* OA models are currently being used, none of them can comprehensively mimic all facets of OA including the interplay of cartilage and subchondral bone injury/degeneration and synovial inflammation [28]. *In vitro* OA models can be grouped according to cell-extracellular matrix (ECM) arrangement into monolayer, scaffold-free or scaffold-based 3D models and cartilage explants of which explant cultures provide the closest approximation of in vivo conditions, making it possible to study cells in their natural ECM and to observe matrix degeneration and repair [29–31].

Currently, OA-like processes are induced *in vitro* either mechanically by static or dynamic compression or chemically by supplementation of the culture medium with cytokines [32–39]. However, these one-dimensional injury models do not address the full complexity of the OA pathophysiology described above. Furthermore, in order to provide a valid OA model the role of the synovial membrane and subchondral bone and their interaction with chondrocytes and the cartilage matrix needs to be taken into account [32, 33].

To date no model has incorporated a co-culture of all three main tissues involved in OA, cartilage, subchondral bone and synovium with a multimodal approach to induce OA combining chemical and mechanical injury. Therefore, the model validated in the current study aims to address all major key components of naturally occurring osteoarthritis by establishing an osteochondral-synovial membrane explant co-culture, in which we induced OA-like changes through addition of IL1β and TNFα combined with a partial-thickness cartilage defect, a typical result of mechanical injury [40].

We hypothesized that: (1) Combination of a partial-thickness defect in the osteochondral explant with supplementation of the culture medium with IL1β and TNFα will lead to an inflammatory reaction and cartilage degeneration similar to naturally occurring OA. (2) Co-culturing synovial tissue explants and osteochondral explants will augment the induced inflammatory response and lead to more marked changes in ECM composition and OA-like changes than osteochondral explant culture alone. (3) Induction of inflammation (addition of IL1β and TNFα and creation of a partial-thickness defect) will cause a shift of synoviocytes to M1 type macrophages in the synovial tissue explants.

## Methods

### Cartilage explants

Osteochondral explants (n = 18 per horse) were aseptically harvested from three adult horses (age 11y, 17y and 18y) without any clinical history of joint disease. These were hospital patients euthanized for reasons unrelated to the current study (septic peritonitis, laminitis and ileus).

All samples were collected within three hours post mortem. Following arthrotomy, the articular cartilage, synovium and joint capsule were visually inspected to ensure no macroscopic lesions were present. Osteochondral explants, 6mm in diameter, were harvested from the medial femoral condyle, using a commercially available, sterilized hollow punch. Synovial membrane explants of approximately 6mm diameter were harvested from the radiocarpal joint, which proved to be an easily accessible source for a synovial membrane explant of standardized size.

### Explant culture, osteoarthritis model

The osteochondral and synovial membrane explants were washed with phosphate buffered saline (PBS, Lonza, Basel, Switzerland) three times prior to further processing and were randomly allocated to one of four experimental groups (n = 6 explants/group): osteochondral explant without injury (control-1), osteochondral–synovial membrane explant co-culture without injury (control-2), osteochondral explant with injury (OA-model-1) or osteochondral–synovial membrane explant co-culture with injury (OA-model-2).

Explants were cultured in serum free Dulbecco's Modified Eagle's Medium (DMEM, Lonza) with 1% penicillin,1% streptomycin, 1% Insulin,-Transferrin-Selenium-Ethanolamine (ITS-X, ThermoFischer Scientific, Massachusetts, USA) and 50μg/ml L-proline (Carl Roth, Karlsruhe, Germany) for up to three weeks. In the OA-model groups 10ηg/ml recombinant human IL1β and TNFα (ImmunoTools, Friesoythe, Germany) were added to the cell culture medium and a 2mm diameter, partial thickness cartilage defect was created in the center of the osteochondral explant using a commercially available biopsy punch (Biopsy Punch, Stiefel, Munich, Germany) to induce inflammation. Culture medium was changed twice a week.

Explants and cell culture supernatant were harvested at week1, week2 and week3 for analysis by histology and immunohistochemistry, qPCR and ELISA, with 2 technical replicates/ biological replicate/time point/analysis method for all groups.

### Histology, immunohistochemistry

For histology, osteochondral explants were fixed in 4% buffered formalin [41], decalcified in 8% neutral EDTA [41], embedded in paraffin and 4 μm-thick sections were mounted on APES-glutaraldehyde-coated slides (Sigma-Aldrich, St. Louis, Missouri, USA). Consecutive sections were stained with Hematoxylin and Eosin (H&E) and Safranin-O [41].

For immunohistochemistry, sections were deparaffinized, rehydrated and endogenous peroxidase was blocked with 0.6% hydrogen peroxide in methanol (15 min at room temperature). Nonspecific binding of antibodies was prevented by incubation with 1.5% normal goat serum (Dako Cytomation, Santa Clara, California, USA) in PBS (30 min at room temperature). Immunohistochemistry staining was carried out for Col1, Col2, MMP1, MMP3, MMP13, ADAMTS5 and IL6. For collagen type-1 (Col1) and Col2 staining, pre-treatment by digestion in 0.02% hyaluronidase (Sigma-Aldrich) for 4 hours and 0.1% pepsin in 0.01N HCL (Sigma-Aldrich) for 30 minutes at 37°C was necessary. Antigen heat retrieval by pre-treatment in 65°C waterbath for 120 min in in Tris-EDTA-buffer (pH 9.0) was necessary for MMP1 and in 0.01 M citrate buffer (pH 6.0) for MMP3, MMP13 and ADAMTS5. For IL6, antigen retrieval was performed by pre-treatment (in 80°C waterbath for 120 min) in Tris-EDTA-buffer (pH 9.0) for 2 hours.

**Fig 1:**
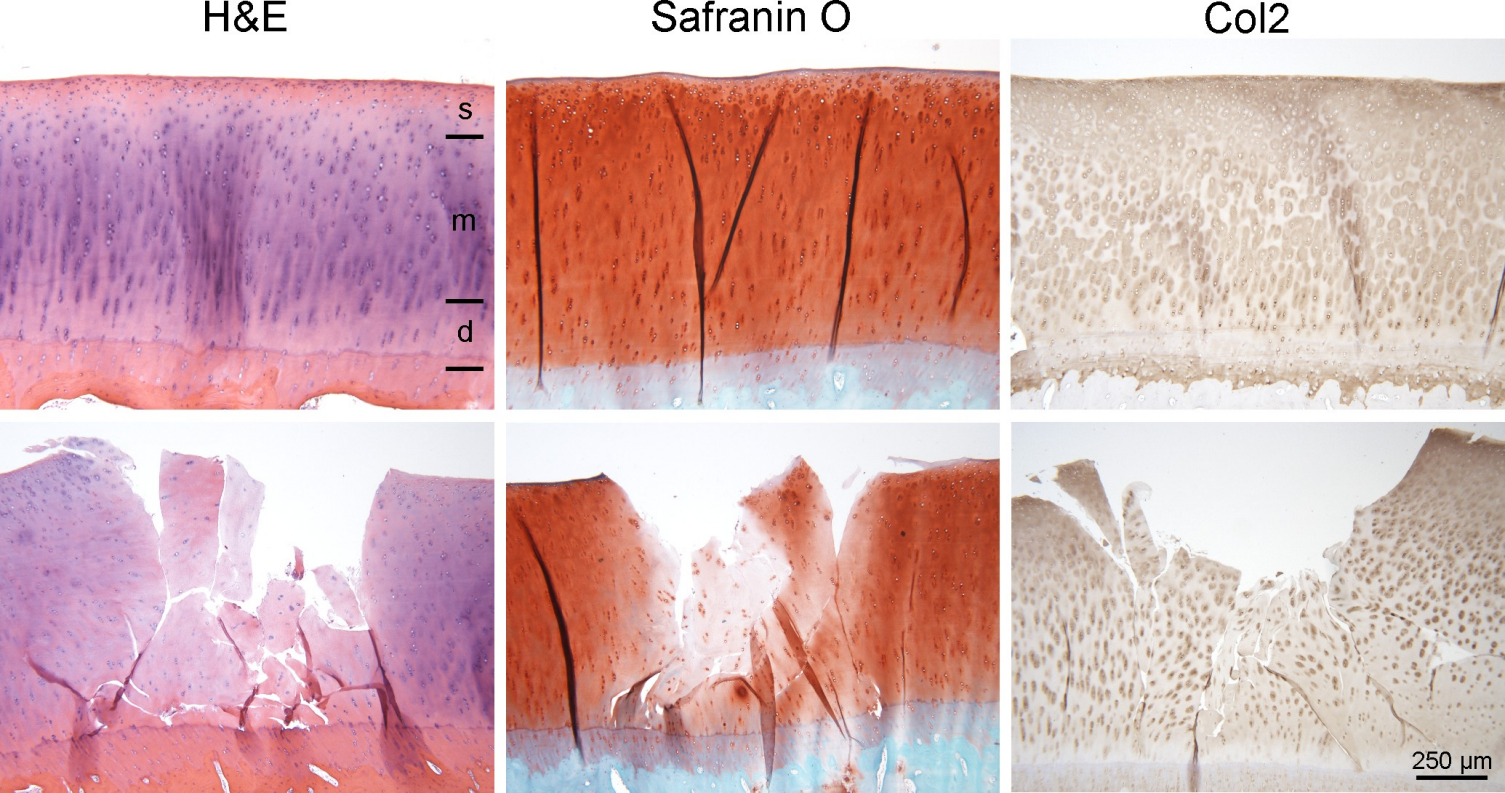
H&E staining, Safranin O staining and immunohistochemical staining for Col2. Osteochondral explants of control groups (top row) and OA model groups (bottom row) after Hematoxylin and Eosin (H&E) staining, Safranin O staining, and immunohistochemical staining for collagen type-2 (Col2) at week 3. For further analysis, the cartilage explants were divided into three zones (see top left micrograph): superficial zone (s), middle zone (m) and deep zone (d).

Primary antibodies (S1 Table) were incubated overnight at 4°C. An appropriate BrightVision peroxidase system (Immunologic) was used and peroxidase activities were localized with Diaminobenzidine (Sigma-Aldrich). Cell nuclei were counterstained with Mayer’s hematoxylin. For analysis of Col1 and Col2 content and enzyme or cytokine activity, cartilage explants were divided into three zones: superficial zone, middle zone and deep zone (Fig 1). For each zone positive cells were counted in three fields of vision at 40x magnification. The percentage of positive cells per zone was calculated for each time point, group and horse.

### Quantitative PCR

After harvesting of the explants, the cartilage layer was removed from the subchondral bone plate at the level of the calcified cartilage layer. Cartilage discs were then snap frozen in liquid nitrogen and stored at -80°C until analysis.

Frozen cartilage discs were pulverized using a Biopulverizer (BioSpec, Bartlesville, Oklahoma, USA) which had prior been cooled in liquid nitrogen for one minute and then transferred to an Eppendorf tube. For 10mg of tissue, 500μl of RNA isolation reagent (PureZOL, Bio-Rad, Hercules, California, USA) were added to the powdered cartilage tissue. The sample was vortexed for 5 minutes and incubated on ice for 30 minutes. After the removal of the tissue debris, the supernatant was transferred to a new Eppendorf tube and 100μl of chloroform (Sigma-Aldrich) were added. After the phase separation, the aqueous phase was collected and total RNA was recovered by the addition of isopropyl alcohol (Sigma-Aldrich) and glycerol (Thermo Scientific). The mixture was incubated for 20 minutes on ice and centrifuged for 30 minutes 13000 rpm. Subsequently, the total RNA pellet was washed twice with 75% EtOH and solubilized in RNase-free water. Genomic DNA was removed by Ambion DNA-free, DNA removal kit (Life Technologies, Carlsbad, California, USA) for 30 minutes at 37°C.

Gene expression was analyzed for the ECM genes Col1, Col2, Col10 and Aggrecan (Acan) and the inflammatory marker genes MMP1, MMP3, MMP13, ADAMTS5, IL6. All primers were designed using Primer3 software. Specificity of the primers was analyzed using the NCBI primer blast tool and in silico PCR tool with the UCSC genome browser. The primer sequences are shown in S2 Table.

25ng of RNA from each sample was used for the qPCR reaction. RevTrans QPCR One-Step EvaGreen kit (Bio&Sell, Feucht, Germany) was used for the PCR reaction, for cDNA synthesis and subsequently for the qPCR reaction according to the user manual. The reaction mixtures were incubated for 15 minutes at 50°C for cDNA generation, followed by the qPCR reaction: 95°C for 5 minutes, 95°C for 15 seconds, 55°C for 20 seconds and 72°C for 30 seconds. For each gene, a reaction mixture without the total RNA template was run as a negative control. The transcript data was analyzed using Agilent AriaMx 1.1 software (Agilent Technologies, Santa Clara, California, USA). The transcript level for the genes of interest was normalized to the transcription level of glyceraldehyde-3-phosphate dehydrogenase (GAPDH) and presented as a relative transcript to GAPDH.

### Enzyme-linked immunosorbent assay (ELISA)

Concentrations of MMP1, MMP3, MMP13, IL6 (Cloud Clone Corp, Katy, Texas, USA) and ADAMTS5 (MyBioSource, San Diego, California, USA) in the cell culture supernatant were determined using commercially available ELISA kits according to the instructions provided by the manufacturer. Values are presented in pg/mL. A standard curve was calculated for each assay with the following detection limits: MMP1 < 0.134ng/mL, MMP3 < 12.9pg/mL, MMP13 < 0.29ng/mL, ADAMTS5 = 31.2ng/mL and IL-6 <3pg/ml.

### Nitrite oxide, urea

Based on their arginine metabolism via nitric oxide synthase to NO and citrulline or via arginase to ornithine and urea [19], macrophages were classified into M1 respectively M2 macrophage by measuring NO and urea levels in the cell culture supernatant of control group 2 and OA model group 2 using commercially available assays (QuantiChrom Urea Assay Kit, BioAssay Systems, Hayward, California, USA and Griss Reagent System, Promega, Madison, Wisconsin, USA) according to manufacturer’s instructions. As cartilage does not contain macrophages and chondrocytes do not produce urea [42], NO and urea levels were not measured in control group 1 or OA model group 1. The concentration of nitrite in the samples was calculated using a standard curve constructed with NaNO_2_. The concentration of urea in the samples was calculated according to the standard curve obtained with known concentrations of a urea (50–1,600 μM). To determine the relationship between type 1 (M1) and type 2 (M2) macrophages in the synovial explant we calculated the NO (μM)/urea (μM) ratio [43, 44].

### Statistical analysis

Statistical analyses were performed using R version 2.4.1 (https://cran.r-project.org). We applied linear mixed effects model with function lmer in package lme4 with fixed effects for treatment and time point. A random intercept effect for horse was modelled to account for the covariance structure in our data caused by technical replicates for each time-point and repeated measurements from week 1–3 for each of the 3 biological replicates. Our data met the assumptions for linear mixed effects models. Residuals and random effects of horse were normally distributed and residuals showed variance homogeneity for all our analyses. Post hoc analysis was performed using pairwise comparisons of groups and time points with p<0.05 regarded significant after multiple testing correction applying Tukey's HSD using R package lsmeans v2.27–61 [45].

## Results

### Histology and immunohistochemistry

Overall cells in the osteochondral explants and in the synovial membrane explants remained viable over the entire cell culture period based on their histological appearance. A decrease in staining intensity could be seen within the defect and adjacent to the defect in OA-model-1 and 2 on H&E, Safranin-O and Col2 stained sections (Fig 1) at all time points. Cartilage residues in the defect area showed a decrease in cellularity.at all time points. No decrease in stain uptake could be detected in the samples without defects (control-1 and 2).

**Fig 2:**
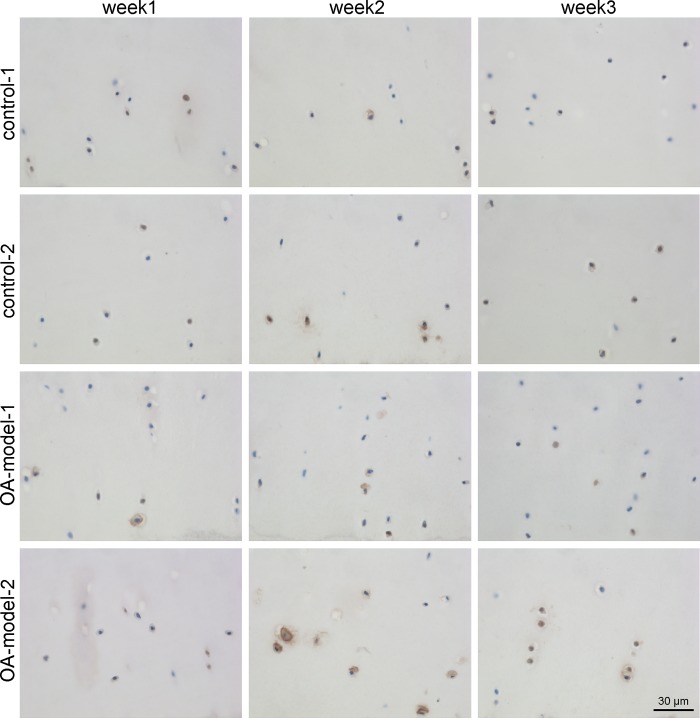
Representative micrographs showing immunohistochemical staining of MMP13 in the middle zone of osteochondral explants over time. The percentage of positive cells per zone was calculated for each time point, group and horse. Increase of MMP13 positive chondrocytes was detected in OA-model-1 compared to control-1 and in week 3.

There was no difference in overall Col1 and Col2 staining over time or between the four groups. The mean percentage of cells stained positively for MMP1, MMP 3, MMP13, ADAMTS5 and IL-6 as well as the significances between the groups and different time points are displayed in Table 1. There were no significant differences between the 2 control groups. The two OA groups differed only in their MMP1 expression with OA-model-2 showing a significantly higher number of MMP1 positive cells than OA-model-1. OA-model-1 showed a significantly higher number of MMP3, MMP13 (Fig 2) and IL6 positive cells compared to control-1. OA-model-2 showed a significantly higher number of positively stained cells compared to control-2 for MMP1 and MMP3.

**Table 1:** Contrasts, lsmean (%positive cells), SE and significances of Immunohistochemistry for MMP1, MMP3, MMP13, ADAMTS5 and IL-6. Values for statistical analysis were calculated from mean values of the three cartilage zones (superficial, middle, deep, detailed in S3 Table). Significant values are highlighted in bold font.

	contrast	lsmean	SE	*p-*value
**MMP1**	OA-model-1 vs control-1	12.7 vs 4.9	2.7	0.19
	OA-model-2 vs control-2	24.7 vs 12.3	2.7	**0.01**
	control-1 vs control-2	4.9 vs -12.3	2.7	0.24
	OA-model-1 vs OA-model-2	12.7 vs 24.7	2.7	**0.02**
	week1 vs week2	0.3 vs 10.8	2.4	**0.009**
	week2 vs week3	10.8 vs 29.9	2.4	**<0.0001**
	week1 vs week3	0.3 vs 29.9	2.4	**<0.0001**
**MMP3**	OA-model-1 vs control-1	13.3 vs 6.9	1.7	**0.005**
	OA-model-2 vs control-2	17.3 vs 11.6	1.7	**0.01**
	control-1 vs control-2	6.9 vs 11.6	1.7	0.05
	OA-model-1 vs OA-model-2	13.3 vs 17.3	1.7	0.11
	week1 vs week2	4.3 vs 12.1	1.5	**<0.0001**
	week2 vs week3	12.1 vs 20.4	1.5	**<0.0001**
	week1 vs week3	4.3 vs 20.4	1.5	**<0.0001**
**MMP13**	OA-model-1 vs control-1	31.4 vs 24.7	2.4	**0.04**
	OA-model-2 vs control-2	33 vs 27.3	2.4	0.15
	control-1 vs control-2	24.7 vs 27.3	2.4	0.68
	OA-model-1 vs OA-model-2	31.4 vs 33	2.4	0.92
	week1 vs week2	25.7 vs 29.2	2.1	0.21
	week2 vs week3	29.3 vs 32.4	2.1	0.29
	week1 vs week3	25.7 vs 32.4	2.1	**0.007**
**ADAMTS5**	OA-model-1 vs control-1	48.2 vs 38.03	4.2	0.1
	OA-model-2 vs control-2	47.2 vs 39.7	4.2	0.3
	control-1 vs control-2	38.03 vs 39.7	4.2	0.98
	OA-model-1 vs OA-model-2	48.2 vs 47.2	4.2	0.99
	week1 vs week2	37.1 vs 46.2	3.7	0.05
	week2 vs week3	46.2 vs 46.6	3.7	0.99
	week1 vs week3	37.1 vs 46.5	3.7	**0.04**
**IL-6**	OA-model-1 vs control-1	13.1 vs 3.8	3.3	**0.04**
	OA-model-2 vs control-2	14.1 vs 9.6	3.3	0.54
	control-1 vs control-2	3.8 vs 9.6	3.3	0.32
	OA-model-1 vs OA-model-2	13.1 vs 14.1	3.3	0.99
	week1 vs week2	7.3 vs 11.8	2.9	0.26
	week2 vs week3	11.8 vs 11.4	2.9	0.32
	week1 vs week3	7.3 vs 11.4	2.9	0.98

**Fig 3:**
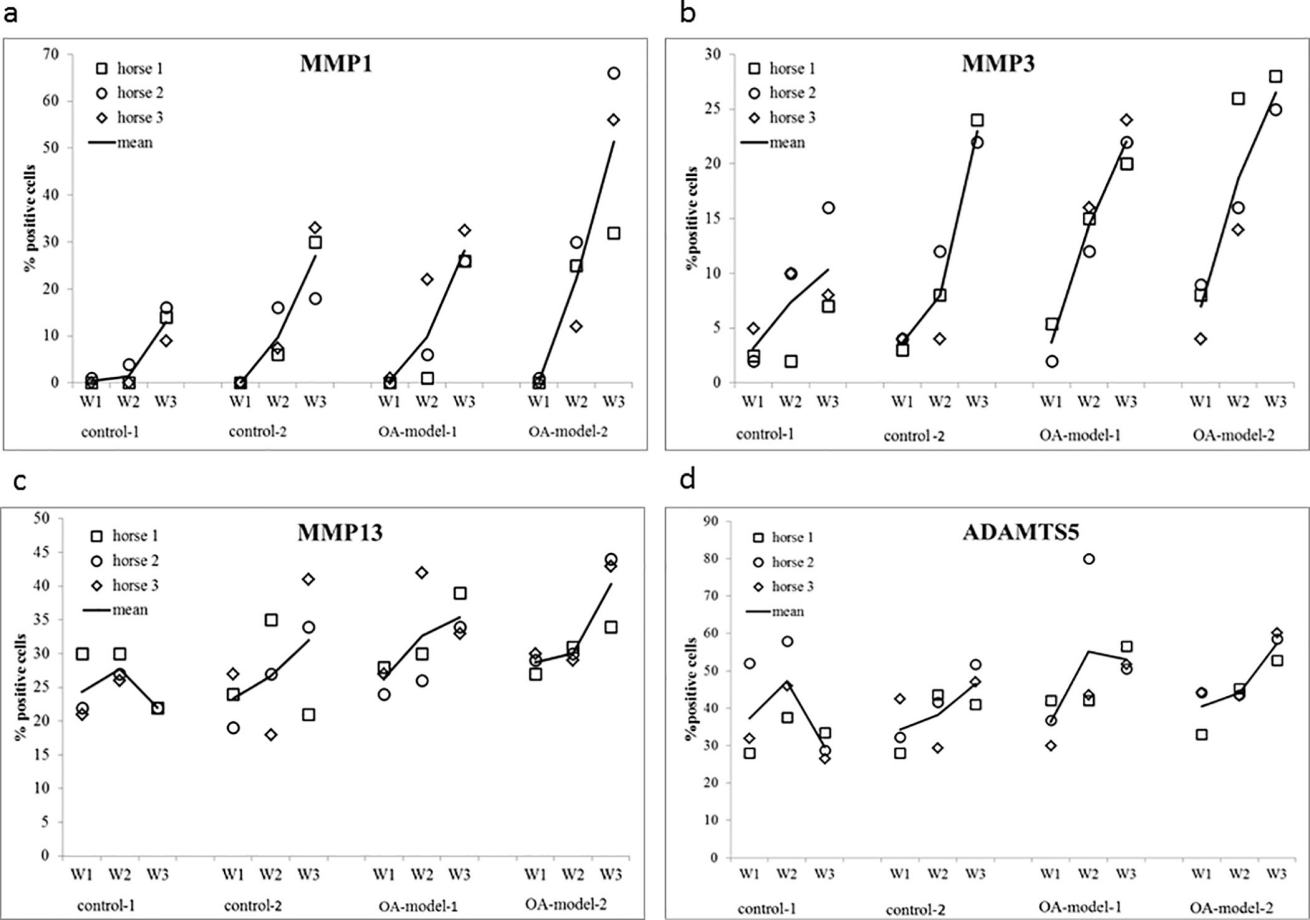
**Dot Plots showing the mean percentage of positive stained (IHC) cells of all three zones for a) MMP1, b) MMP3, c) MMP13 and d) ADAMTS5 for each individual horse for control groups (control 1, control 2) and OA-model groups (OA-model 1, OA-model 2) at different time points (week1 (W1), week 2 (W2), week 3 (W3)).** Mean values of the three horses shown are displayed as a line.

For MMP1 and MMP3 a significant increase in percentage of positive cells over time (week 1 to week 2 and week 2 to week 3) could be detected in all four groups (Table 1, Fig 3A and 3B). MMP13 and ADAMTS5 showed a significant increase between week 1 and week 3 (Fig 3C and 3D).

Patterns and mean values of MMP1, MMP3, MMP13. ADAMTS5 and IL-6 staining in different cartilage zones over time are displayed in S3 Table.

### qPCR

Gene expression mean values of the ECM genes Col1, Col2, Col10 and Acan and the inflammatory marker genes MMP1, MMP3, MMP13, ADAMTS5 and IL6 as well as significances between the 4 groups and different time points are displayed in Table 2. There were no significant differences between the 2 control groups with the exception of their Col2 expression, which was significantly higher in control-2 compared to control-1. The two OA groups showed no significant differences, neither in their ECM nor their inflammatory marker genes. OA-model-1 showed a significantly higher expression of inflammatory markers MMP1, MMP3, MMP13 and IL6 compared to control-1. OA-model-2 showed a significantly higher expression of ECM gene Col2 and a significantly higher expression of inflammatory markers MMP1, MMP3, MMP13 and IL6 compared to control-2.

**Table 2:** Contrasts, lsmean, SE and significanes of mRNA expression relative to GAPDH (ct) for the 4 different groups (control-1, control-2, OA-model-1, OA-model-2) and 3 different time points (week 1, week 2, week 3) for Col1, Col2, Col10, Acan, MMP1, MMP3, MMP 13, ADAMTS5 and IL-6. Significant values are highlighted in bold font.

	contrast	lsmean (ct)	SE	*p-*value
**Col1**	OA-model-1 vs control-1	38.1 vs 36.9	0.78	0.50
	OA-model-2 vs control-2	37.5 vs 35.9	0.78	0.25
	control-1 vs control-2	38.1 vs 37.5	0.78	0.92
	OA-model-1 vs OA-model-2	36.9 vs 35.9	0.78	0.68
	week1 vs week2	35.5 vs 35.1	0.69	0.81
	week2 vs week3	35.1 vs 40.7	0.69	**<0.0001**
	week1 vs week3	35.5 vs 40.7	0.69	**<0.0001**
**Col2**	OA-model-1 vs control-1	29.1 vs 28.8	0.32	0.95
	OA-model-2 vs control-2	27.6 vs 29.4	0.32	**0.0009**
	control-1 vs control-2	29.1 vs 27.6	0.32	**0.0075**
	OA-model-1 vs OA-model-2	28.8 vs 29.4	0.32	0.63
	week1 vs week2	28.1 vs 28.7	0.28	0.22
	week2 vs week3	28.7 vs 29.3	0.28	0.38
	week1 vs week3	28.1 vs 29.3	0.28	**0.009**
**MMP1**	OA-model-1 vs control-1	27.4vs 30.9	0.43	**<0.0001**
	OA-model-2 vs control-2	27vs 30.3	0.43	**<0.0001**
	control-1 vs control-2	30.9 vs 30.3	0.43	0.46
	OA-model-1 vs OA-model-2	27.4 vs 27	0.43	0.83
	week1 vs week2	28.4 vs 28.9	0.40	0.40
	week2 vs week3	28.9 vs 29.5	0.40	0.40
	week1 vs week3	28.4 vs 29.5	0.40	**0.03**
**MMP3**	OA-model-1 vs control-1	25.5vs 28.3	0.33	**<0.0001**
	OA-model-2 vs control-2	26.04 vs 28.5	0.33	**<0.0001**
	control-1 vs control-2	28.3 vs 28.5	0.33	0.96
	OA-model-1 vs OA-model-2	25.5 vs 26.0	0.33	0.68
	week1 vs week2	27.4 vs 27.2	0.29	0.88
	week2 vs week3	27.2 vs 26.6	0.29	0.16
	week1 vs week3	27.4 vs 26.6	0.29	0.35
**MMP13**	OA-model-1 vs control-1	26.3 vs 29.8	0.47	**<0.0001**
	OA-model-2 vs control-2	27.3 vs 29.9	0.47	**0.001**
	control-1 vs control-2	29.8 vs 29.9	0.47	0.99
	OA-model-1 vs OA-model-2	26.3 vs 27.3	0.47	0.29
	week1 vs week2	28.3 vs 27.6	0.42	0.41
	week2 vs week3	27.6 vs 29.1	0.42	**0.013**
	week1 vs week3	28.3 vs 29.1	0.42	0.24
**ADAMTS 5**	OA-model-1 vs control-1	34.3 vs 34.7	0.44	0.77
	OA-model-2 vs control-2	33.9vs 34.6	0.44	0.42
	control-1 vs control-2	34.7 vs 34.6	0.44	0.99
	OA-model-1 vs OA-model-2	34.3 vs 33.9	0.44	0.84
	week1 vs week2	33.3 vs 34.2	0.40	0.09
	week2 vs week3	34.2 vs 35.6	0.40	**0.0037**
	week1 vs week3	33.3 vs 35.6	0.40	**<0.0001**
**IL-6**	OA-model-1 vs control-1	27.7 vs 31.7	0.54	**<0.0001**
	OA-model-2 vs control-2	27.2 vs 31.9	0.54	**<0.0001**
	control-1 vs control-2	31.7 vs 31.9	0.54	0.99
	OA-model-1 vs OA-model-2	27.7 vs 27.2	0.54	0.84
	week1 vs week2	28.9 vs 29.2	0.57	0.89
	week2 vs week3	29.2 vs 30.7	0.57	**0.022**
	week1 vs week3	28.9 vs 30.7	0.57	**0.004**
**Col10**	OA-model-1 vs control-1	31.9 vs 32.5	0.54	0.31
	OA-model-2 vs control-2	32.3 vs 31.9	0.54	0.72
	control-1 vs control-2	32.5 vs 32.3	0.54	0.35
	OA-model-1 vs OA-model-2	31.9 vs 32.3	0.54	0.68
	week1 vs week2	32.5 vs 31.7	0.35	0.06
	week2 vs week3	31.7 vs 32.2	0.35	0.24
	week1 vs week3	32.5 vs 32.2	0.35	0.70
**Acan**	OA-model-1 vs control-1	29.9 vs 30.1	0.61	0.99
	OA-model-2 vs control-2	30.1 vs 28.9	0.61	0.27
	control-1 vs control-2	30.1 vs 28.9	0.61	0.29
	OA-model-1 vs OA-model-2	29.9 vs 30.1	0.61	0.99
	week1 vs week2	29.9 vs 29.5	0.53	0.81
	week2 vs week3	29.5 vs 29.9	0.53	0.81
	week1 vs week3	29.9 vs 29.9	0.53	1.00

**Fig 4:**
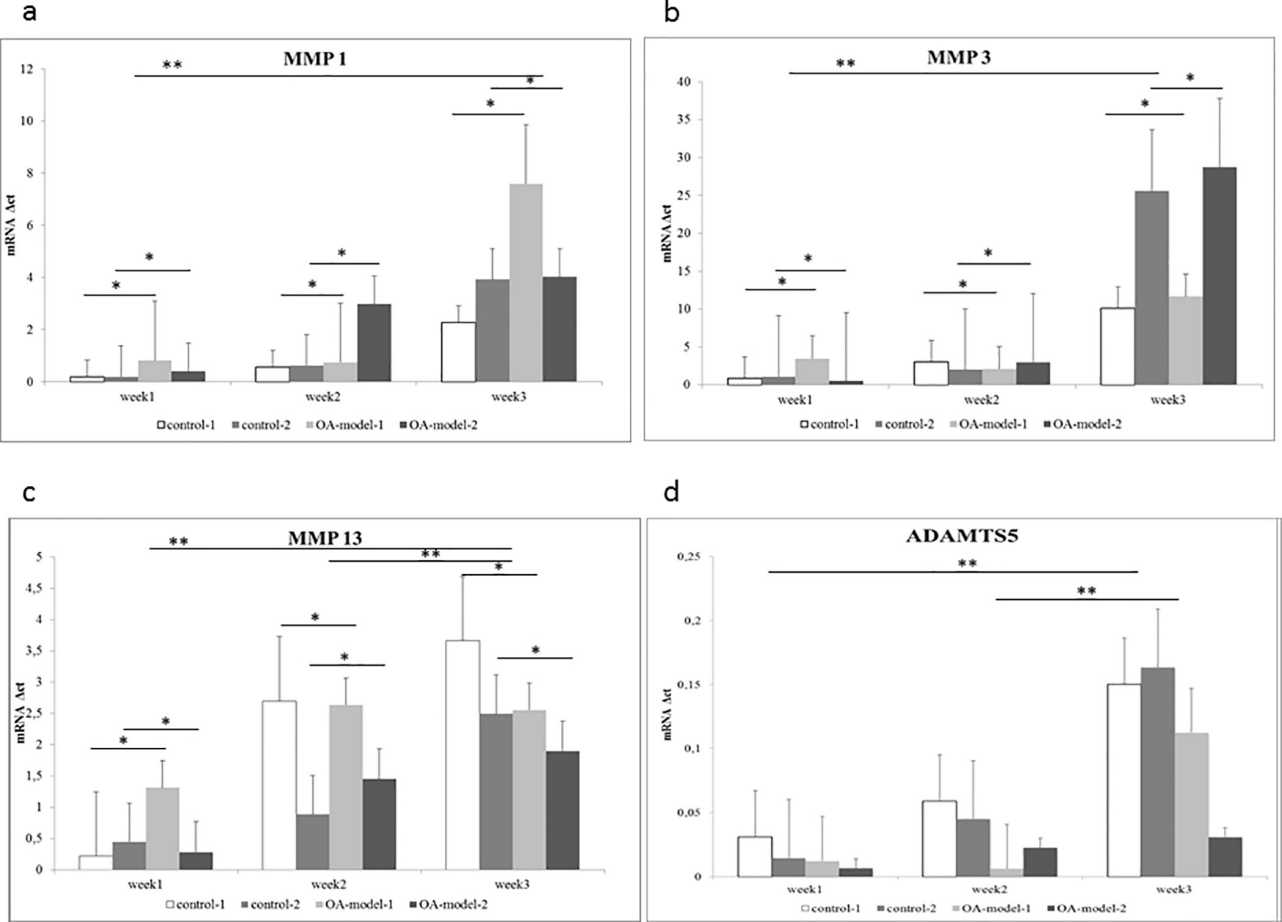
**mRNA expression relative to GAPDH (Δct) for a)MMP1, b)MMP3, c)MMP13 and d)ADAMTS5 for control groups (control-1, control-2) and OA models (OA-model-1, OA-model-2) at week1, week2 and week3.** * *P*<0,05 for differences between the groups; ** *P*< 0,05 for differences between time-points; the individual *P*-values are displayed in Table 2.

Gene expression for Col1 and Col2 was significantly downregulated between weeks 1 and 3 and for Col1, the decrease was also significant between weeks 2 and 3. MMP1, ADAMTS5 and IL6 were significantly downregulated between weeks 1 and 3, MMP13, ADAMTS5 and IL6 also showed a significant downregulation between weeks 2 and 3 (Fig 4).

### ELISA

Concentrations of MMP1, MMP3, MMP13, ADAMTS5 and IL6 in the cell culture supernatant and the significances between the 4 groups and different time points are displayed in Table 3. MMP1, MMP3, MMP13, ADAMTS5 and IL6 concentrations showed no significant differences between the OA groups, between the OA and control groups and amongst the control groups except for a significantly higher IL6 expression in control-1 versus both control-2 and OA-model-1. However, a significant decrease in MMP1 antigen-concentration was found between week1 and week2 (and week1 and3. in all groups. Also, a significant decrease in MMP3 antigen concentration was found between week1 and week3 and week2 and week3 in all groups (Table 3).

**Table 3:** Contrasts, lsmean, SE and significanes of ELISA for the 4 different groups (control-1, control-2, OA-model-1, OA-model-2) and 3 different time points (week 1, week 2, week 3) for MMP1, MMP3, MMP 13, ADAMTS5 and IL-6. Significant values are highlighted in bold font.

	contrast	lsmean	SE	*p-*value
**MMP1**	OA-model-1 vs control-1	-0.43 vs -0.45	0.08	0.99
	OA-model-2 vs control-2	-0.32 vs -0.32	0.08	1.00
	control-1 vs control-2	-0.45 vs -0.32	0.08	0.40
	OA-model-1 vs OA-model-2	-0.43 vs -0.32	0.08	0.48
	week1 vs week2	-0.01 vs -0.51	0.07	**<0.0001**
	week2 vs week3	-0.51 vs -0.63	0.08	0.23
	week1 vs week3	-0.01 vs -0.63	0.08	**<0.0001**
**MMP3**	OA-model-1 vs control-1	2.12 vs 2.82	0.28	0.09
	OA-model-2 vs control-2	2.51 vs 3.10	0.30	0.23
	control-1 vs control-2	2.82 vs 3.10	0.30	0.79
	OA-model-1 vs OA-model-2	2.12 vs 2.51	0.27	0.48
	week1 vs week2	3.16 vs 2.70	0.25	0.18
	week2 vs week3	2.70 vs 2.05	0.25	**0.03**
	week1 vs week3	3.16 vs 2.05	0.26	**0.0005**
**MMP13**	OA-model-1 vs control-1	-0.20 vs 0.14	0.22	0.39
	OA-model-2 vs control-2	-0.11 vs -0.02	0.27	0.97
	control-1 vs control-2	0.14 vs -0.02	0.26	0.94
	OA-model-1 vs OA-model-2	0.20 vs 0.11	0.23	0.98
	week1 vs week2	0.18 vs -0.12	0.19	0.31
	week2 vs week3	-0.12 vs -0.18	0.21	0.95
	week1 vs week3	0.18 vs -0.18	0.21	0.20
**ADAMTS5**	OA-model-1 vs control-1	1.93 vs 1.95	0.02	0.68
	OA-model-2 vs control-2	1.89 vs 1.92	0.02	0.54
	control-1 vs control-2	1.95 vs 1.92	0.02	0.30
	OA-model-1 vs OA-model-2	1.93 vs 1.89	0.02	0.20
	week1 vs week2	1.93 vs 1.93	0.21	0.99
	week2 vs week3	1.93 vs 1.91	0.21	0.62
	week1 vs week3	1.93 vs 1.91	0.21	0.64
**IL-6**	OA-model-1 vs control-1	0.99 vs 1.67	0.17	**0.002**
	OA-model-2 vs control-2	1.11 vs 1.15	0.18	0.99
	control-1 vs control-2	1.67 vs 1.15	0.18	**0.03**
	OA-model-1 vs OA-model-2	0.99 vs 1.11	0.17	0.91
	week1 vs week2	1.36 vs 1.13	0.14	0.28
	week2 vs week3	1.13 vs 1.20	0.15	0.89
	week1 vs week3	1.36 vs 1.20	0.15	0.56

Table 4 summarizes the differences in IHC, qPCR and ELISA measurements between OA-model groups and OA-model groups and their corresponding controls.

**Table 4:** Statistically significant differences between the control and OA-model groups analysed by Immunohistochemistry (IHC), qPCR and ELISA.

IHC		MMP1	MMP3	MMP13	ADAMTS5	IL-6
	OA-model-1 vs control-1	**-**	**+**	**+**	**-**	**+**
	OA-model 2 vs control-2	**+**	**+**	**-**	**-**	**-**
	OA-model 1 vs OA-model-2	**+**	**-**	**-**	**-**	**-**
	control-1 vs control-2	**-**	**-**	**-**	**-**	**-**
qPCR						
	OA-model-1 vs control-1	**+**	**+**	**+**	**-**	**+**
	OA-model 2 vs control-2	**+**	**+**	**+**	**-**	**+**
	OA-model 1 vs OA-model-2	**-**	**-**	**-**	**-**	**-**
	control-1 vs control-2	**-**	**-**	**-**	**-**	**-**
ELISA						
	OA-model-1 vs control-1	**-**	**-**	**-**	**-**	**+**
	OA-model 2 vs control-2	**-**	**-**	**-**	**-**	**-**
	OA-model 1 vs OA-model-2	**-**	**-**	**-**	**-**	**-**
	control-1 vs control-2	**-**	**-**	**-**	**-**	**+**

+ difference between groups

- no difference between groups

### Nitrite oxide, urea

**Fig 5:**
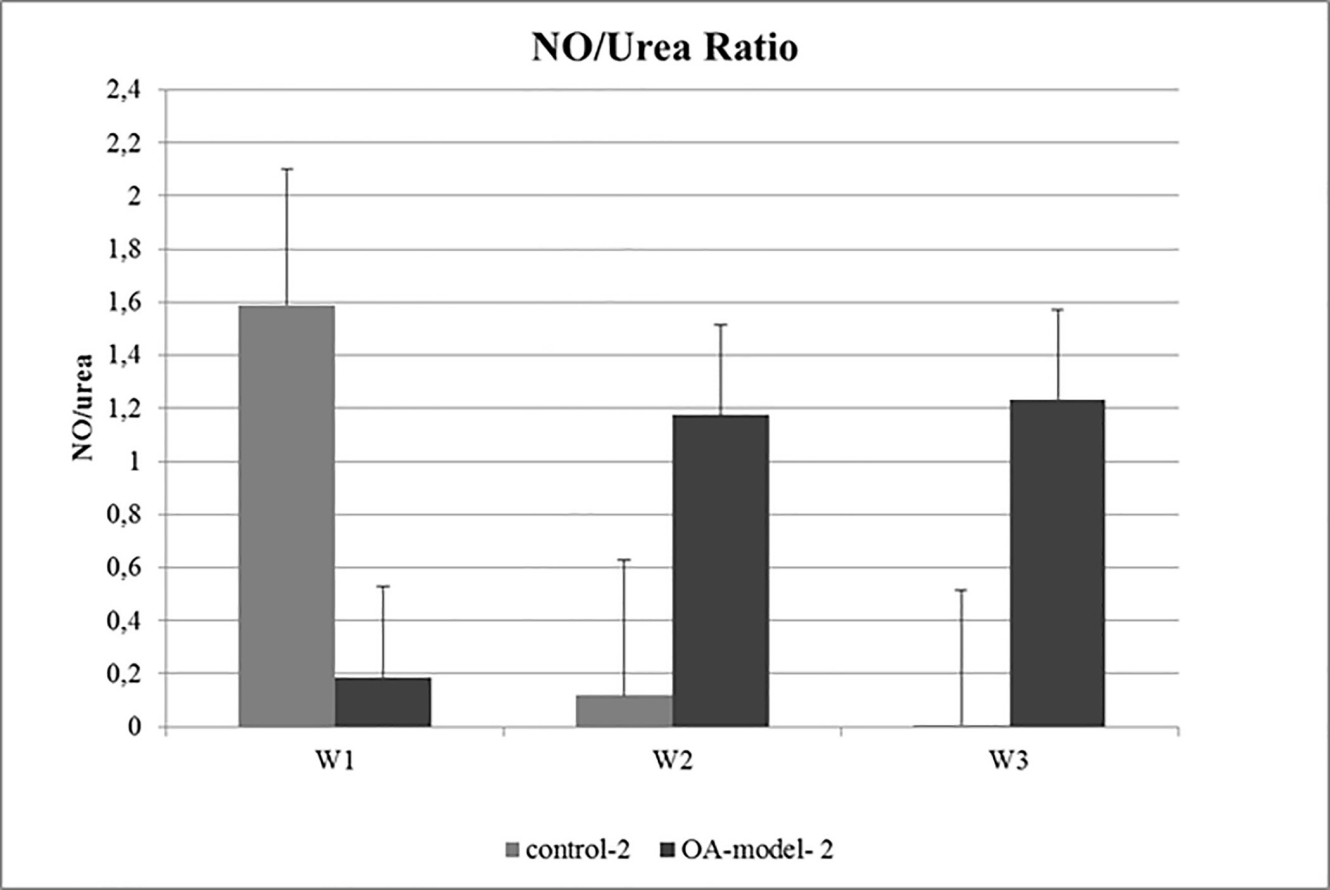
Mean NO/urea ratio of OA-model-2 and control-2 at week1 (W1), week2 (W2) and week3 (W3). Control-2 showed a decrease in NO/urea ratio from week1 to week3, whereas OA-model-2 showed an increase from W1 to W2 and W3 indicating a shift of synovial macrophages toward the pro-inflammatory M1 phenotype.

Calculation of the NO/urea ratio revealed a higher NO/urea ratio in OA-model-2 compared to control-2 indicating a higher percentage of pro-inflammatory M1 macrophages in the OA-model-2 group (Fig 5, S4 Table).

## Discussion

Since the combination of the poor self-healing capacity of articular cartilage and the lack of disease-modifying therapies has created a large clinical and socioeconomic demand, well-characterized models of OA are necessary to elucidate the key signaling pathways and molecular switches of OA pathophysiology and to identify specific therapeutic targets. In order to develop a pathophysiologically relevant *in vitro* model for OA which reflects the complexity of the disease characterised by a vicious circle of cartilage degeneration accompanied by synovial inflammation and remodelling of the subchondral bone, we established an osteochondral-synovial membrane explant co-culture and induced OA-like changes by adding IL1β and TNFα to the culture medium to induce inflammation and creating a partial thickness cartilage defect to simulate this common sequela of mechanical cartilage injury.

We demonstrated good viability of the osteochondral and synovial explants throughout the 21-day culture period based on histology, qPCR and ELISA. Explants produced steady levels of ECM-associated mRNA and secreted measurable quantities of cytokines throughout the culture period indicating good cell viability.

Moreover, control groups showed no significant differences in gene expression for ECM markers (Col1, Col10 and Acan) suggesting maintenance of ECM integrity throughout the culture time. Expression of Col2 was significantly increased in control-2 versus control-1 as well as in OA-model-2 compared to control-2, which may suggest a more pronounced change in ECM production in the osteochondral-synovial explant co-culture OA model compared to the osteochondral explant alone.

qPCR showed a significant increase in gene expression of proteolytic enzymes MMP1, MMP3, and MMP13 as well as IL6 in the OA model groups compared to the control groups. These results could be partly strengthened by immunohistochemistry: In both OA model groups a significant increase in MMP3 positive cells could be observed over time when compared to the corresponding control groups. In MMP1 only OA-model-2 showed a significant increase in positive stained cells compared to control-1. In contrast, only OA-model-1 was significantly different to control-1 in IL-6 and MMP13 expression. Interestingly we observed increased MMPs and ADAMTS5 expression in the control groups as well as the OA-model groups, however this is consistent with previous studies showing upregulation of MMPs, especially MMP3, and ADAMTS5 also in healthy cartilage in vivo and *in vitro* [37, 46–48]. The current study confirmed these findings also for ADAMTS5 measured by qPCR, Elisa and immunohistochemistry. A relatively constant number of ADAMTS5 positive stained cells (about 70%) were found in immunohistochemistry in all four groups at the three different time points in the calcified zone. Moreover no significant differences were found between groups for ELISA and qPCR. In accordance with previous studies [37, 46–48], which found ADAMTS5 to play a role not only in Acan degradation in naturally occurring OA but also in physiologic cartilage turnover, in our study Acan levels remained constant despite the increased number of ADAMTS5 positive cells, indicating that no cartilage breakdown was initiated by the MMP and ADAMTS5 expression in the control groups.

MMP13 gene expression profile showed a statistically non-significant trend to decrease in the osteochondral-synovial explant co-culture compared to osteochondral explants alone (Fig 4), which is consistent with previous studies also documenting a reduced magnitude of increase of MMP13, IL6 and ADAMTS4 gene expression in osteochondral-synovial explant co-cultures compared to osteochondral explant monocultures [48, 49]. This was enforced by results of immunohistochemistry in the current study showing no significant difference (i.e. a less pronounced increase) in number of IL-6 and MMP13 positive cells comparing OA-model-2 and control-2. Combined with the significantly decreased Col2 expression in control-1 compared with control-2, this finding might support the suggestion by previous studies that co-culture also has a protective effect on cartilage degradation by decreasing the expression of ECM collagenases such as MMP13 [48].

Recently the role of macrophage like synoviocytes in the progression of OA was investigated by several studies. It was reported that M1 macrophages play a key role in promotion of catabolic processes and inflammation in naturally occurring OA as well as *in vitro* experiments [11–18, 21]. M1 synovial macrophages release pro-inflammatory cytokines namely IL1β and TNFα, proteolytic enzymes (MMP1, MMP3, MMP13, ADAMTS5) and reactive oxygen species (nitric oxide) contributing to joint degeneration in OA [11–18, 21]. M1 macrophages are possibly polarised from macrophages in synovial explants [14] or recruited from a different source in the culture (cartilage, subchondral bone). To further investigate the potential influence of the synovial membrane explant on changes in ECM contents and expression of proteolytic enzymes and IL6 in our co-culture OA model we calculated the NO/urea ratio in the supernatant of OA-model-2 and control-2 as previously described [43, 44]. Analysis revealed, that the activity of M1 synovial macrophages increased over time in the OA-model-2 compared to the respective control-2. This result suggests a shift of synovial macrophages towards M1 phenotype in the synovial membrane triggered by IL1β and TNFα reflecting the natural situation as encountered in osteoarthritic joints.

Overall results of the current study suggest that the proposed osteochondral explant model is a valid *in vitro* model for OA. Based on histology, qPCR and ELISA, we could demonstrate good viability of the osteochondral and synovial explants of both control and OA groups throughout the 21-day culture period. Combining cytokine-induced inflammation through TNFα and IL1β with a partial thickness cartilage defect to simulate this common sequela of mechanical cartilage injury elicited an OA-like inflammatory response in both proposed models to a similar extent. However, we observed additional pathologies associated with naturally occurring OA, such as a more marked reaction including a greater decrease in Col2, a different pattern of MMP13 and a shift of synovial macrophages toward M1 phenotype in the co-culture with synovial membrane explants. Therefore addition of another joint-tissue to the OA-model potentially offers a wider spectrum of pathologies associated with OA for future analysis.

## Supporting information

S1 Table Sources and dilutions of the antibodies used for IHC staining for Col1, Col1, MMP1, MMP3, MMP13, ADAMTS5 and IL-6. (DOCX)

S2 Table Primer sequences used for qPCR for Col1, Col2, ColX, Acan, MMP1, MMP3, MMP13, ADAMTS5 and IL-6. (DOCX)

S3 Table Mean number (%) of positive cells per cartilage zone for IHC staining for MMP1, MMP3, MMP13, ADAMTS5 and IL6 in week 1 (W1), week 2 (W2) and week 3 (W3) for all 4 groups. Numbers of positive cells (%) were obtained by counting 3 fields of vision at 40x magnification per zone at each time point. Mean numbers (%) were calculated thereafter.
(DOCX)

S4 Table NO/urea (mean, SD) ratio of OA-model-2 and control-2 at week1, week2 and week3. (DOCX)
