# Supplementary material for: Co-culture of osteochondral explants and synovial membrane as in vitro model for osteoarthritis
Source: PLoS One. 2019 Apr 2;14(4):e0214709. doi: 10.1371/journal.pone.0214709 (PMC6445514; doi:10.1371/journal.pone.0214709)
Supplement: S1 Table — (DOCX) [file pone.0214709.s001.docx]

**S1 Table.** Sources and dilutions of the antibodies used for IHC staining for Col1, Col1, MMP1, MMP3, MMP13, ADAMTS5 and IL-6.

| **Antibody** | **Source** | **Clone** | **Dilution** |
| --- | --- | --- | --- |
| Collagen type I | Southern Biotech | poly | 1:100 |
| Collagen type II | Thermofischer Scientific | 2B1.5 | 1:100 |
| MMP1 | Santa Cruz | poly | 1:600 |
| MMP3 | Neo Markers | poly | 1:50 |
| MMP13 | Thermofischer Scientific | poly | 1:50 |
| ADAMTS5 | Novus Biologicals | poly | 1:300 |
| IL6 | R&D Systems | poly | 1:100 |
