# Supplementary material for: Co-culture of osteochondral explants and synovial membrane as in vitro model for osteoarthritis
Source: PLoS One. 2019 Apr 2;14(4):e0214709. doi: 10.1371/journal.pone.0214709 (PMC6445514; doi:10.1371/journal.pone.0214709)
Supplement: S2 Table — (DOCX) [file pone.0214709.s002.docx]

**S2 Table.** Primer sequences used for qPCR for Col1, Col2, ColX, Acan, MMP1, MMP3, MMP13, ADAMTS5 and IL-6.

| **Gene** | **Species** | **Forward Primer** | **Reverse Primer** |
| --- | --- | --- | --- |
| Col1a2 | Equine | TCCATCTGGAGAGCCTGGTA | CACCTGGTAGACCACGTTCA |
| Col2a1 | Equine | CCTTGGTGGAAACTTTGCTG | CCTTGCATTACTCCCATCTG |
| MMP1 | Equine | CAGTGCCTTCAGAAACACGA | GCTTCCCAGTCACTTTCAGC |
| MMP3 | Equine | TGTGGAGGTGATGCACAAATC | GCATGCCAGGAAATGTAGTGAA |
| MMP13 | Equine | TGGTCCAGGAGATGAAGACC | GATGGCATCAAGGGATAAGG |
| ADAMTS5 | Equine | GCAAGTGTGTGGACAAAACC | TCTGGGAGCAGGATTATTGC |
| IL6 | Equine | ATGGCAGAAAAAGACGGATG | GGGTCAGGGGTGGTTACTTC |
| Col10 | Equine | TACAGGCATAAAAGGGCCAC | CCAGGAACACCTTGTTCTCC |
| Aggrecan | Equine | CTATGAGGACGGCTTCCACC | GAAACTCATCCTTGTCCCCA |
