# Supplementary material for: Co-culture of osteochondral explants and synovial membrane as in vitro model for osteoarthritis
Source: PLoS One. 2019 Apr 2;14(4):e0214709. doi: 10.1371/journal.pone.0214709 (PMC6445514; doi:10.1371/journal.pone.0214709)
Supplement: S3 Table — Numbers of positive cells (%) were obtained by counting 3 fields of vision at 40x magnification per zone at each time point. Mean numbers (%) were calculated thereafter. (DOCX) [file pone.0214709.s003.docx]

**S3 Table.** Mean number (%) of positive cells per cartilage zone for IHC staining for MMP1, MMP3, MMP13, ADAMTS5 and IL6 in week 1 (W1), week 2 (W2) and week 3 (W3) for all 4 groups. Numbers of positive cells (%) were obtained by counting 3 fields of vision at 40x magnification per zone at each time point. Mean numbers (%) were calculated thereafter.

|  |  | **MMP1** | | | **MMP3** | | | **MMP 13** | | | **ADAMTS 5** | | | **IL6** | | |
| --- | --- | --- | --- | --- | --- | --- | --- | --- | --- | --- | --- | --- | --- | --- | --- | --- |
| groups* | zones | W1 | W2 | W3 | W1 | W2 | W3 | W1 | W2 | W3 | W1 | W2 | W3 | W1 | W2 | W3 |
| control-1 | superficial | 1.6 | 6 | 1.6 | 3 | 8 | 5 | 21 | 6 | 10 | 35 | 30 | 0 | 0 | 3 | 5 |
|  | middle | 0 | 0 | 18 | 3.6 | 6 | 14 | 12 | 19 | 20 | 29 | 44 | 37 | 0 | 4 | 7 |
|  | deep | 0 | 0 | 6.6 | 1 | 10 | 5 | 63 | 73 | 40 | 22 | 36 | 48 | 0 | 3 | 20 |
| control-2 | superficial | 0 | 10 | 0 | 3 | 0 | 0 | 5 | 0 | 3 | 3 | 0 | 5 | 5 | 15 | 3 |
|  | middle | 0 | 13 | 27 | 1 | 10 | 32 | 14 | 30 | 30 | 33 | 49 | 58 | 5 | 18 | 5 |
|  | deep | 0 | 0 | 40 | 1.6 | 10 | 20 | 70 | 43 | 53 | 70 | 43 | 58 | 11 | 22 | 0 |
| OA-model-1 | superficial | 1.6 | 33 | 50 | 0 | 5 | 0 | 12 | 3 | 3 | 0 | 5 | 0 | 3 | 5 | 12 |
|  | middle | 0 | 5 | 26 | 5 | 18 | 29 | 13 | 27 | 21 | 34 | 43 | 42 | 14 | 13 | 6 |
|  | deep | 0 | 0 | 0 | 2 | 13 | 12 | 80 | 80 | 70 | 25 | 43 | 65 | 8 | 0 | 25 |
| OA-model-2 | superficial | 1.6 | 16 | 0 | 1.6 | 20 | 12 | 11 | 3 | 3 | 0 | 0 | 5 | 3 | 5 | 5 |
|  | middle | 0 | 24 | 53 | 11 | 20 | 35 | 13 | 26 | 20 | 36 | 46 | 55 | 9 | 23 | 14 |
|  | deep | 0 | 23 | 82 | 0 | 23 | 0 | 80 | 80 | 70 | 80 | 77 | 80 | 5 | 33 | 33 |
