# Supplementary material for: Co-culture of osteochondral explants and synovial membrane as in vitro model for osteoarthritis
Source: PLoS One. 2019 Apr 2;14(4):e0214709. doi: 10.1371/journal.pone.0214709 (PMC6445514; doi:10.1371/journal.pone.0214709)
Supplement: S4 Table — (DOCX) [file pone.0214709.s004.docx]

**S4 Table.** NO/urea (mean, SD) ratio of OA-model-2 and control-2 at week1, week2 and week3.

|  | **control-2** | **OA-model2** |
| --- | --- | --- |
| week 1 | 1.59 (0.84) | 0.19 (1.08) |
| week 2 | 0.12 (0.09) | 1.18 (0.05) |
| week 3 | 0.01 (0.01) | 1.23 (0.30) |
